# Numerical Modeling of Ink Widening and Coating Gap in Roll-to-Roll Slot-Die Coating of Solid Oxide Fuel Cell Electrolytic Layer

**DOI:** 10.3390/polym12122927

**Published:** 2020-12-07

**Authors:** Seongyong Kim, Jongsu Lee, Minho Jo, Changwoo Lee

**Affiliations:** 1 Department of Mechanical Design and Production Engineering, Konkuk University, 120, Neungdong-ro, Gwangjin-gu, Seoul 05029, Korea; arsen6788@konkuk.ac.kr (S.K.); als8080@konkuk.ac.kr (M.J.); 2 Department of Printed Electronics Engineering, Sunchon National University, 255, Jungang-ro, Jeollanam-do, Suncheon 57922, Korea; jslee0505@scnu.ac.kr; 3 Department of Mechanical Engineering, Konkuk University, 120, Neungdong-ro, Gwangjin-gu, Seoul 05029, Korea

**Keywords:** flexible functional device, thin film coating, slot-die, surface tension, coating gap

## Abstract

Slot-die coatings are advantageous when used for coating large-area flexible devices; in particular, the coating width can be controlled and simultaneous multi-layer coatings can be processed. To date, the effects of ink widening and the coating gap on the coating thickness have only been considered in a few studies. To this end, we developed two mathematical models to accurately estimate the coating width and thickness that consider these two effects. We used root mean square deviation (RMSD) to experimentally verify the developed method. When the coating gap was increased, the coating width increased and the coating thickness decreased. Experimental results showed that the estimated performances of the coating width and thickness models were as high as 98.46% and 95.8%, respectively. We think that the developed models can be useful for determining the coating conditions according to the ink properties to coat a functional layer with user-defined widths and thicknesses in both lab- and industrial-scale roll-to-roll slot-die coating processes.

## 1. Introduction

The roll-to-roll manufacturing (R2R) process has become a subject of interest because of its low cost and mass production capability [1]. With this process, large-area functional layers can be fabricated on a flexible polymer substrate using various printing or coating techniques, such as blade coating, spray coating, and slot-die coating [2,3,4]. In particular, slot-die coating provides the advantages of controlling the coating width by changing the design of a shim plate assembled in the slot-die chamber and simultaneously processing multi-layer coatings. Unlike blade coating or spray coating, slot-die coating produce a coat with predictable thickness through process condition control, and because it is a noncontact coating method, it has excellent surface roughness. These can contribute to a decrease in the production costs of flexible electronic devices. Thus, slot-die coating is a good candidate for coating large-area flexible devices on polymer substrates [5,6], and numerous research groups have studied the fabrication of such devices, namely photovoltaic and fuel cells [7,8,9].

Recently, an electrolytic layer, which is a separator in a solid oxide fuel cell (SOFC), was fabricated using the R2R slot-die coating process. Many studies have analyzed ink behavior in the slot-die coating process and reported the fabrication of single and multi-functional layers using a slot-die coater. In particular, Ruschak et al. [10] established the desired ranges for coating parameters, such as the coating speed, the surface tension on ink, and the pressure applied to the inlet and outlet of the coating bead, to obtain a high-quality slot-die coating. Lee et al. [11] analyzed the effects of the geometry of a slot-die coater and the coating gap on the coating thickness. Ning et al. [12] studied the change in the coating quality according to the concentration of polymer particles in a coated solution. They determined that the maximum coating speed is dependent on the polymer concentration. Cavalho et al. studied the correlations among the coating gap, Reynolds number, and capillary number, which are determined by ink properties such as the density, surface tension, and viscosity of the solution. Yang et al. [13] found a thicker coated layer when the density and viscosity of the coated solution were high. Lee et al. [14] proposed a method to prevent cracks during the brittle electrolytic layer coating using the R2R process. Park et al. [15] developed a technique to alleviate a pinned edge in the coated layer in the R2R slot-die coating process. Kim et al. [16] analyzed the ink velocity profile at the tip of a slot-die coater using finite element analysis and suggested a desired geometry of the same for improving the uniformity of the coating layer. From previous studies, the behavior of the slot-die coater has been studied using volumetric [17] and viscocapillary models [18]. The volumetric model is based on the continuity equation between the tip of the slot-die coater and the surface of the substrate, which estimates the thickness of the coated layer according to the layer geometry and flow rate of an ink supplier, such as the mono and syringe pump. The effects of the ink widening determined by its surface tension, surface energy of the substrate, and coating gap, however, are not considered. On the contrary, in the viscocapillary model, the minimum permissible coating thickness that can form a stable coating bead can be estimated by considering the ink properties and the coating gap. This model can be used in low-viscosity solution coatings; however, an actual wet coating thickness cannot be estimated. To accurately estimate the thickness of a coated layer, the effects of ink widening and the coating gap on the coating thickness should be considered. However, only a few studies have considered these two effects when estimating the coating thickness in the slot-die coating process. In this paper, we propose an advanced model based on the volumetric model that can estimate the width and thickness of the coated layer considering the aforementioned effects. The developed method was experimentally verified using root mean square deviation (RMSD), which is generally used to numerically evaluate the estimation performance of theoretical and experimental models.

## 2. Mathematical Modeling

**Figure 1:**
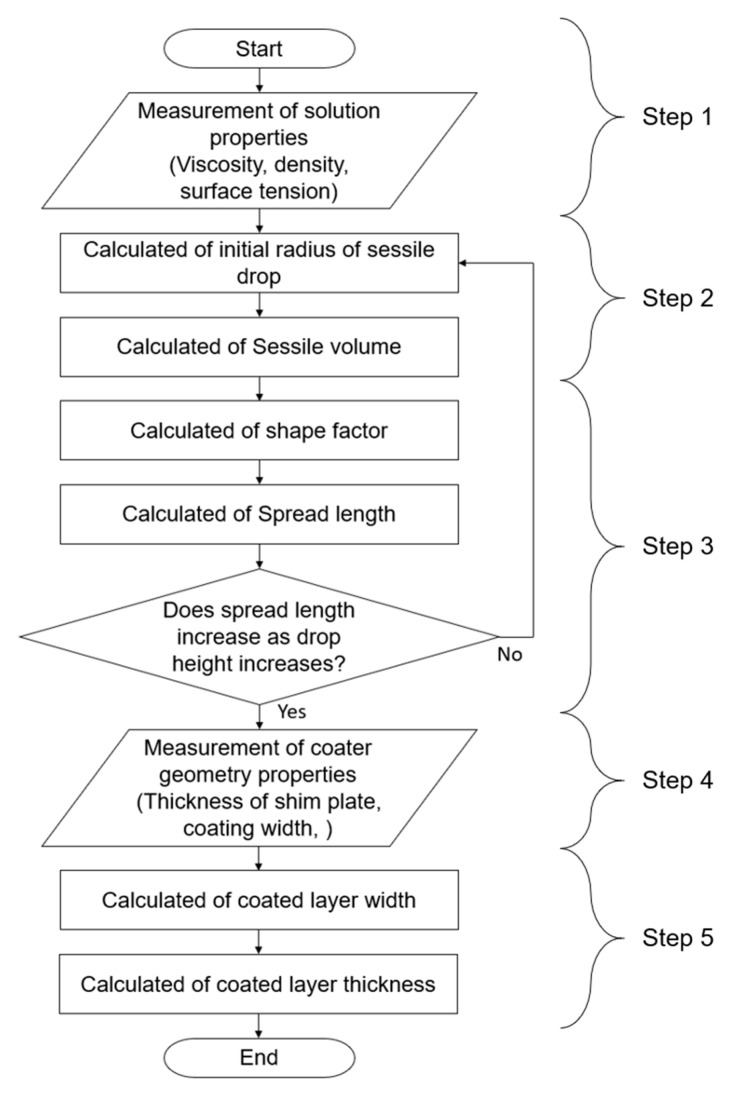
Flow chart for estimating the coated width and thickness using the coating gap and ink properties.

Figure 1 presents the flow chart for estimating the coated width and thickness using the coating gap and ink properties such as density, viscosity, and surface tension. The entire process was carried out in five steps.

**Step 1**. Measure the ink properties: viscosity, density, and surface tension.
**Step 2**. Measure the contact angle in the absence of injection height.
**Step 3**. Calculate the volume of injected ink and the shape factor to determine the widened length of the droplet of ink [19].
**Step 4**. Calculate the widened length and the ratio of the change of the droplet radius of ink deposited on a substrate (*r*(*t*) − *r*_e_) to the ink droplet radius at 0 mm of injection height (*r*_e_) (named widening ratio, *wr*) using Equations (1) and (2), respectively.
**Step 5**. Calculate the coated layer width and thickness (named coating width and coating thickness, respectively) using Equations (3) and (5), respectively.

**Figure 2:**
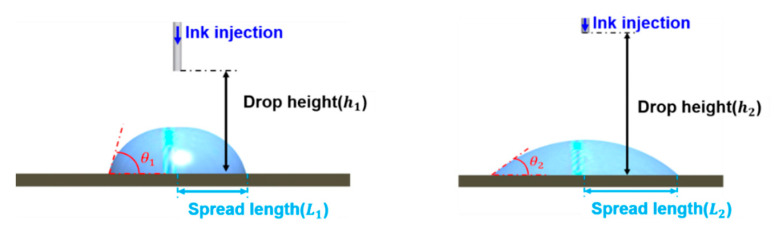
Schematics of the change in the contact angle and coating width by varying the injection height.

**Figure 3:**
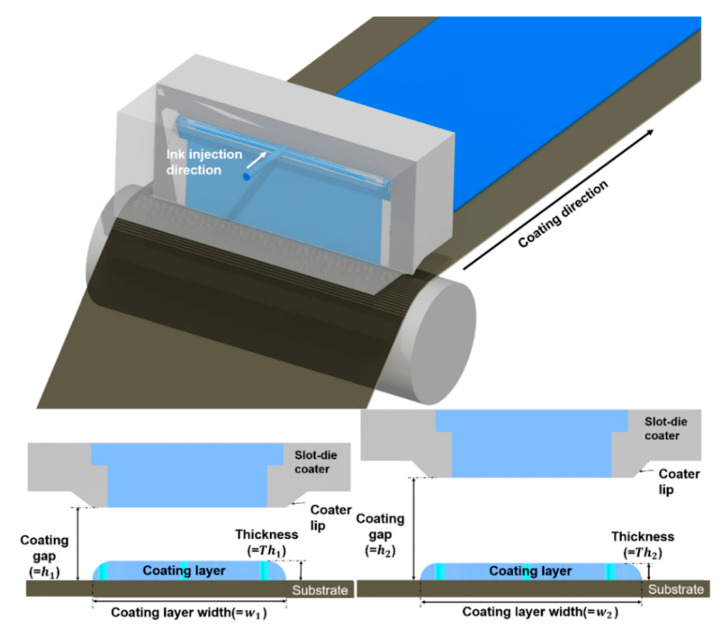
Schematics of the change in the contact angle and coating width by varying the coating gap.

Figure 2 and Figure 3 show schematics of the change in the contact angle and coating width caused by varying the injection height and the coating gap, respectively. Generally, the ink widening in a tensioned web varies according to the injected volume of ink, the injection height, and the surface tension [20]. The droplet radius of the ink according to the injection height can be obtained using Harth’s model, as shown in Equation (1):

(1) $$r\left(t\right)=r_{e}{\left[1-exp\left(\frac{2\gamma_{L}}{r_{e}{}^{12}}+\frac{\rho g}{9r_{e}{}^{10}}\right)\frac{24\lambda v^{4}\left(t+t_{0}\right)}{\pi^{2}\eta }\right]}^{\frac{1}{6}}$$

where *η* is the ink viscosity, *λ* is the shape factor, *γ* is the surface tension of ink, *v* is the volume of injected ink, *r_e_* is the effective radius of the droplet, *t* is the time interval between the ink injection and ink deposition on the substrate, and *g* and *ρ* are the gravitational acceleration and ink density, respectively.

The widened length of the ink, that is, the increase in the radius of the ink increases with increasing ink density and injection height, which suggests that the coating width changes according to the coating gap, as shown in Figure 3.

Based on Harth’s model, we developed a mathematical model to estimate the coating width. The widening ratio (*wr*) can be expressed as:

(2) $$wr=\frac{r\left(t\right)-r_{e}}{r_{e}}$$

The coating width can be obtained using Equation (3) considering the width of the slot-die coater, which is the same as the coating width in the absence of the widening effect, and the widening ratio obtained by substituting Equation (1) into Equation (2).

The coating width considering the widening effect can be estimated using Equation (3):

(3) $$w\left(h\right)=w_{0}+\left(w_{0}*wr\left(h\right)\right)$$

where *w*_0_ is the width of the slot-die coater and *h* is the coating gap. Equation (4) represents the coating thickness model developed in our previous studies for estimating the coating thickness considering the widening effect [21,22].

(4) $$th_{e,d}=s\left(th_{e,w}\right)=\frac{sKf_{r}}{nwV}$$

where *d* is the thickness of the dried coated layer; *s* is the density of the solute of the ink; *w* is the thickness of the wet coated layer; *f_r_* is the flow rate; *n*, *w*, and *V* are the number of strips of the coated layer, width of the unit strip, and web speed, respectively; and *K* is a dimensionless number expressing the severity of the widening effect. This model is based on the mass conservation law derived between the tip of the slot-die coater and the surface of the substrate. However, we did not consider the effect of the coating gap in our model.

The coating thickness model considering the widening effect and the change in width according to the coating gap can be obtained using Equation (5), which was derived by substituting Equation (3) into Equation (4).

(5) $$th_{e,d}=\frac{sKf_{r}}{nw\left(h\right)v}=\frac{sKf_{r}}{n\left(w_{0}+\left(w_{0}*wr\left(h\right)\right)\right)V}$$

## 3. Experimental Verification

### 3.1. Experimental Conditions

**Figure 4:**
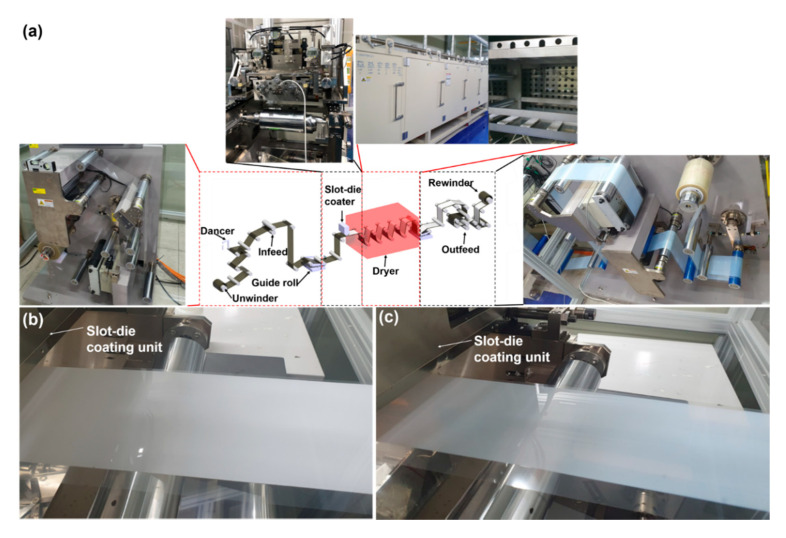
(**a**) The industrial-scale R2R slot-die coating machine in this study, (**b**) the coated layer of YSZ and (**c**) the dielectric layer.

Yttria-stabilized zirconia (YSZ, Sigma Aldrich, St. Louis, MO, USA), an electrolytic layer of an SOFC, and a dielectric layer (BaTiO_3_, Paru Co. Ltd., Suncheon, Korea) were coated using a slot-die coater to experimentally verify the developed coating width and thickness models shown in Equations (3) and (5), respectively. Table 1 shows the properties of YSZ and of the dielectric solution, while Table 2 lists the coating conditions. A mixture of ethanol and toluene (3:7) and acetone were used as the solvents of YSZ and the dielectric solution, respectively. An industrial-scale R2R slot-die coating machine (Toba Co. Ltd., Seoul, Korea), which is shown in Figure 4a, was used to coat the two materials. The tension and web speed were set to 2.7 kgf and 1 m/min, respectively.

### 3.2. Experimental Results

**Figure 5:**
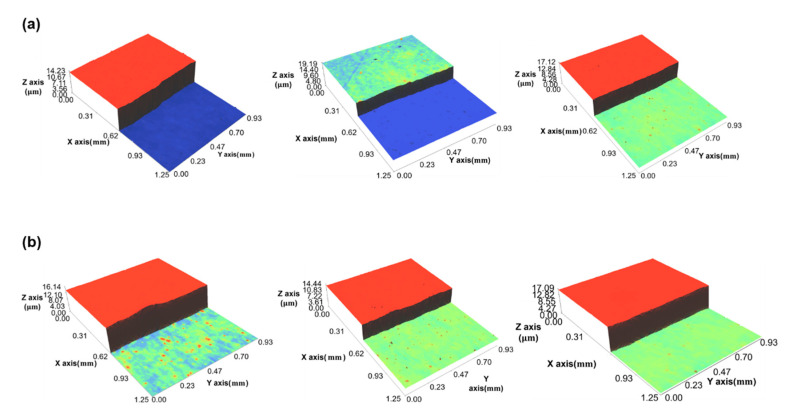
(**a**) YSZ and (**b**) dielectric layers measured using an interferometer.

**Figure 6:**
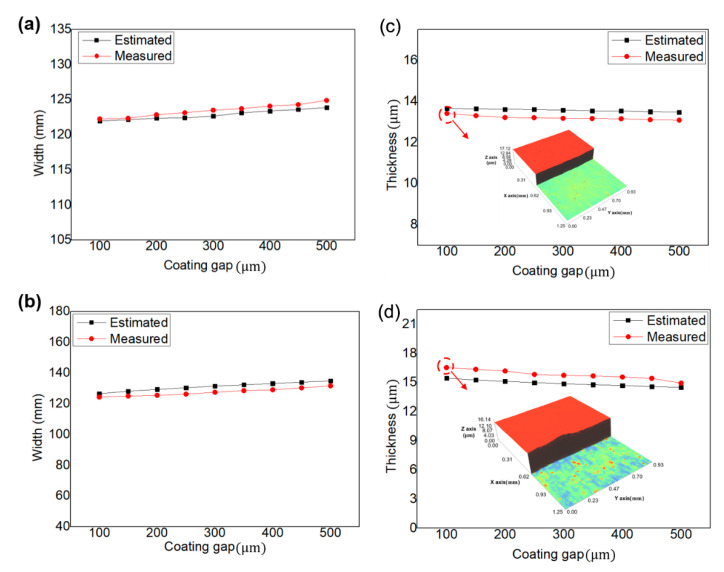
Measured and estimated (**a**) width and (**b**) thickness of the dielectric layer, and measured and estimated (**c**) width and (**d**) thickness of the of the YSZ layer.

Figure 4b,c shows the coated layer of YSZ and the dielectric layer, respectively. Each layer was coated 3 times using 9 different values of coating gaps (100–500 µm with a 50 µm coating gap interval). Figure 5a,b shows the results of measuring the YSZ and dielectric layers using an interferometer (NS-E1000, Nanosystem Co. Ltd., Daejeon, Korea). We compared the measured and estimated thicknesses using the developed models to clearly verify their performance. Figure 6a,b presents the measured and estimated width and thickness of the dielectric layer, respectively, while Figure 6c,d presents the corresponding values of the YSZ layer. It can be seen that the coating width increased and the coating thickness decreased when the coating gap was increased. These results suggest that the widening effect increases with the coating gap. Since the widening effect also increases by increasing the time interval (*t*) between the ink injection and its deposition, it can also increase the width, thereby increasing the coating gap.

The inertia of the ink affects the ink widening more dominantly at higher injection heights. Moreover, the trends of the estimated width and thickness are similar to the measured ones. The estimation performances of the developed models were evaluated using RMSD(Root mean square deviation), which is generally used to numerically evaluate the similarity of approximation values with actual values in the time domain, as shown in Equation (6):

(6) $$MA\left(\%\right)=\sqrt{\frac{\sum_{i=1}^{n}{\left(x_{measured,i}-x_{estimated,i}\right)}^{2}}{n}}$$

where *MA* represents the model accuracy, *n* is the number of the measurements, and *x_measured,i_* and *x_estimated,i_* are the *i*th measured and estimated data, respectively.

**Figure 7:**
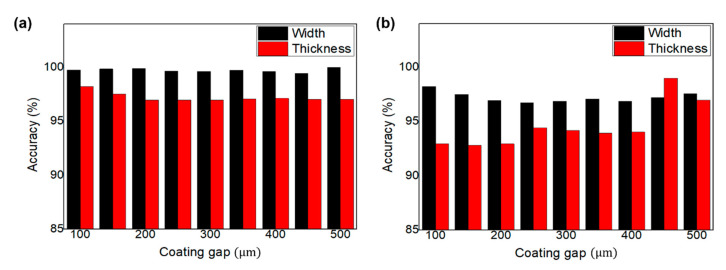
Estimation accuracy of the developed coating width and thickness models for (**a**) the dielectric and (**b**) YSZ layer coating.

Figure 7 presents the estimation accuracy of the developed coating width and thickness models for the dielectric and YSZ layer coating. The average estimation accuracies of the coating width and thickness models are 98.46% and 95.8%, respectively, which demonstrates the superiority of these models. The width of the coated layer was predicted through the spread rate based on the 2D partial wet spreading model, and the thickness was predicted based on the volumetric model. Since the volumetric model cannot consider the thickness in the direction of film transport, the prediction accuracy of thickness is low compared to the width. In addition, the high-viscosity dielectric solution has higher accuracy in the partial wet spreading model.

## 4. Conclusions

We developed two mathematical models to estimate the coating width and thickness considering the coating gap and widening effect of ink in R2R slot-die coatings. We considered the effect of ink properties and its inertia on the coating width and thickness according to the coating gap in our models. We coated the YSZ and dielectric layers using an industrial-scale R2R slot-die coater to experimentally verify the superiority of the developed models, the estimation performances of which were then verified using the RMSD method. Experimental results showed estimation performances of the coating width and thickness models to be 98.46% and 95.8%, respectively. We think the developed model can be useful for determining the coating conditions according to the ink properties to coat a functional layer with user-defined widths and thicknesses in both lab- and industrial-scale R2R slot-die coating processes.

## Figures and Tables

**Table 1:** Properties of YSZ and of the dielectric solution.

Properties	Value	
Solution	Dielectric Solution	YSZ Solution
Radius of sessile ($r_{e}$)	0.167 mm	0.187 mm
Surface tension of solution	26.68 N/mm	28.52 N/mm
Viscosity	0.08 Pa·s	0.03 Pa·s
Weight percent	43.2%	36.8%
Solvent	Acetone	Ethanol 3: Toluene 7

**Table 2:** Coating conditions.

Process Condition	Value
Tension	2.7 kgf
Web speed	1 m/min
Width of Coater	120 mm
Coating gap	100–500 μm
